# Fibroma with minor sex cord elements – an incidental finding in a normal sized ovary A case report with literature review

**DOI:** 10.1186/1746-1596-2-46

**Published:** 2007-12-04

**Authors:** Shramana Mandal, Dipti Mahajan, Somak Roy, Meeta Singh, Nita Khurana

**Affiliations:** 1 Department Of Pathology, Maulana Azad Medical College, New Delhi-110002, India

## Abstract

Ovarian fibroma with minor sex cord element is a rare neoplasm. Microscopically it is composed of predominantly fibromatous or a thecomatous tumor containing scattered minor sex cord elements in less than 10% of the tumor area.

A case of fibroma with minor sex cord elements discovered incidentally in a normal sized ovary in a patient who presented with dysfunctional uterine bleeding is being presented. This is the first case report describing this entity in a normal sized ovary as an incidental finding.

## Background

Ovarian fibroma with minor sex cord elements is a rare entity which was first described by Young and Scully in 1983. Only 8 cases of ovarian fibroma with minor sex cord elements have been reported till date [1-4]. We report a case of fibroma with minor sex cord elements of a normal sized ovary discovered incidentally in a patient who presented with dysfunctional uterine bleeding. This is a rare, distinct clinico-pathologic entity among unclassified sex cord stromal tumors and is the first case report describing, the incidental occurrence of this entity in a normal sized ovary.

## Case Presentation

A 45 year old female, gravida 3, para 3, presented to the gynecology outpatient with complaints of something coming out of the vagina and menorrhagia since the past 6 months. Per speculum examination revealed first degree cervical descent with second degree cystocele and rectocele. On vaginal examination uterus was 6 weeks in size, retroverted and mobile with clear bilateral fornices were free. Abdominal ultrasonography was normal. A clinical diagnosis of dysfunctional uterine bleeding with second degree cervical descent along with second degree cystocele and rectocele was made. Since the patient had completed her family, a total abdominal hysterectomy along with bilateral salpingo-oophorectomy was performed.

**Figure 1:**
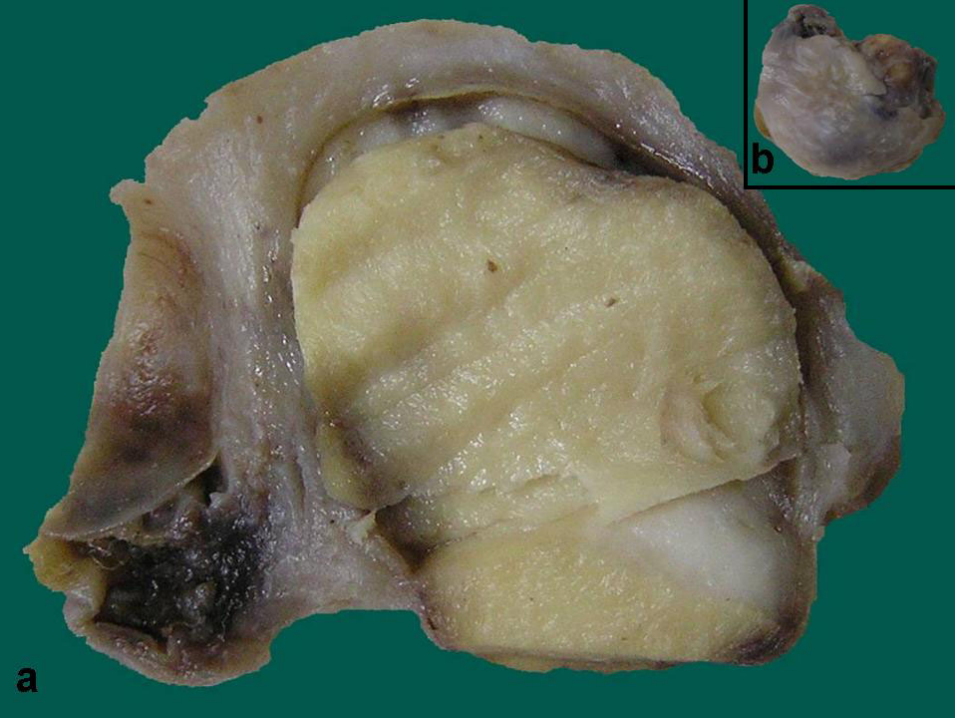
**a)**. Cut surface shows a well circumscribed, yellowish nodule with compressed normal ovarian parenchyma at the periphery. **b)**. Encapsulated normal sized right ovary measuring 2.5 × 2.5 × 1.5 cm

We received a specimen of uterus with cervix along with bilateral adnexa measuring 8 × 3.5 × 2.5 cm. The cervix was everted pearly white in colour with 4 cm long endocervical canal. The endometrium and myometrium thickness were 0.1 cm and 2 cm. The myometrium on serial slicing showed a focus of hemorrhage. The right ovary measured 2.5 × 2.5 × 1.5 cm and was externally covered by capsule. Cut surface of the ovary showed a well circumscribed, yellowish nodular, firm area which measured 1.8 cm in diameter with compressed normal ovarian parenchyma at the periphery (Figure 1). Bilateral fallopian tube and the left ovary were grossly unremarkable.

**Figure 2:**
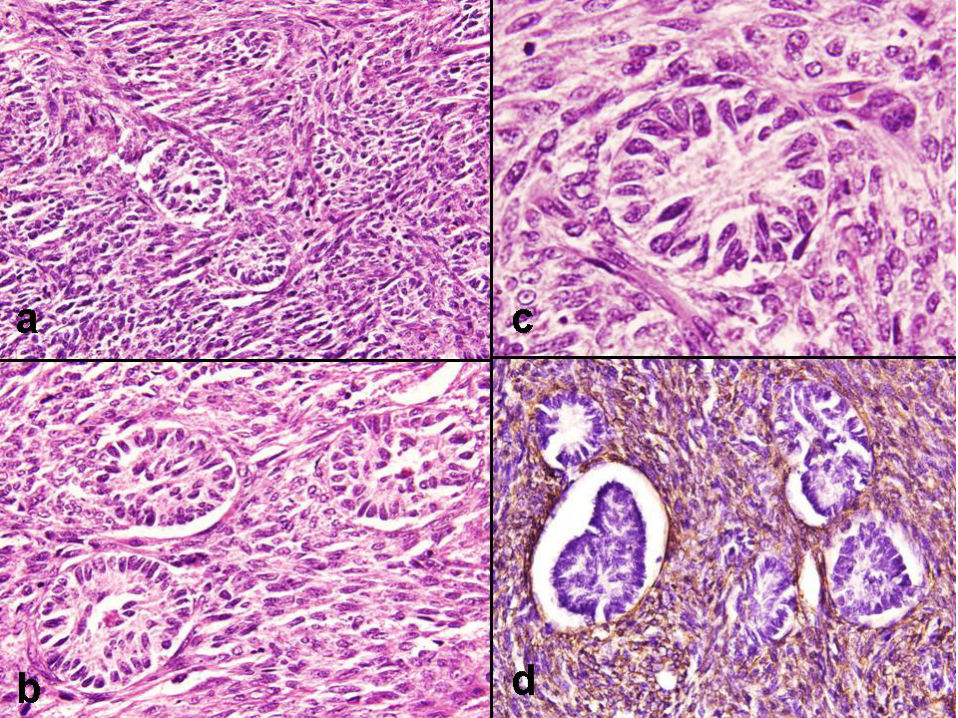
**a)**. Cellular tumor composed of spindled cells in sheets, fascicles and whorls (HE × 200). **b)**. Small aggregates of undifferentiated sex cord type cells forming poorly defined tubular structure (HE × 300). **c)**. Small aggregates were sharply demarcated from the surrounding stroma (HE × 400). **d)**. IHC, SMA intense cytoplasmic staining in the spindled cells (Avidin biotin staining × 400).

Microscopically, sections from the ovary showed a well circumscribed highly cellular tumor composed of spindled cells arranged in sheets, fascicles and whorls (Figure 2a). The cells had scant to moderate amount of eosinophilic cytoplasm with spindled shaped nuclei and 1–2 inconspicuous nucleoli. The cells displayed minimal pleomorphism with 0–1 mitosis per 10 high power fields. Dispersed among these cells were few small aggregates of undifferentiated sex cord type cells with a poorly defined tubular structure (Figure 2b). These small aggregates were sharply demarcated from the surrounding stroma and formed less than 10% of the tumor area (Figure 2c).

The spindled out cells, stained blue in Masson Trichrome stain. Immunohistochemically, the spindled cells were diffusely positive for smooth muscle actin (SMA) and weakly expressed vimentin. These spindled cells were negative for epithelial membrane antigen (EMA) and cytokeratin (Figure 2d). All the cells of the tubular structure were negative for vimentin, SMA and EMA. Based on these above findings a final diagnosis of fibroma with minor sex cord elements was made. The cervix showed chronic inflammation. The endometrium was in proliferative phase with areas of adenomyosis in the myometrium.

## Discussion

Ovarian stromal tumor with minor sex cord elements is a rare neoplasm, which was first described by Young and Scully as a predominantly fibromatous or a thecomatous tumor containing scattered minor sex cord elements in less than 10% of the tumor area [1]. The minor sex cord elements are seen as small nests or tubules of cells resembling granulosa cells, Sertoli cells, or indifferent cells of sex cord type. Only few cases of ovarian stromal tumor with minor sex cord elements have been reported in the literature. To the best of our knowledge only 8 cases of ovarian fibroma with minor sex cord elements have been documented in world literature till date [1-6]. We report the ninth case of ovarian fibroma with minor sex cord elements with review of literature.

The average age of presentation can vary between 16 to 65 years [1]. Our patient was 45 years old. These patients generally present with abdominal pain, bleeding per vaginum and adnexal masses. Our patient also presented with bleeding per vaginum. The tumors can range in size from 1 cm to 10 cm in diameter and can present as an adnexal mass [1]. However, in the present case the ovary was normal in size.

These tumors grossly resemble fibromas or thecomas. They are firm, solid, yellow white to yellow grey in colour. Microscopically they are composed of spindled cells, arranged in intersecting fascicles with variable amount of collagen deposition. The nucleus of the stromal cells is elongated and cigar shaped without prominent nucleoli. The cells of minor sex cord elements resemble granulosa, sertoli, undifferentiated sex cord, or steroid cells. The term "minor" component of sex cord elements is defined as sex cord elements occupying no more than 10% of the area of the tumor on any slide [1]. The individual aggregate of these minor sex cord elements should not be greater than 0.45 mm. Immunihistochemically, the minor sex cord elements are positive for inhibin, calretinin, CD99, CD56, antikeratin antibody KL1 and MIC [2,7].

These tumors have to be differentiated from ovarian fibromatosis, in which there is proliferation of spindled cells with abundant collagen formation and focal areas of edema. The normal follicular structures of the ovary are surrounded by fibrous tissue in fibromatosis whereas in fibroma, the fibrous tissue replaces the ovarian follicles and their derivatives [5,6]. In the present case compressed normal ovary was identified at the periphery and no normal follicular structure could be seen within the fibroma.

The other differential diagnoses include, Brenner tumor and adenofibromas. The epithelial aggregates of Brenner tumor are composed of transitional cells, mucinous cells or both and sometimes have a central lumen containing eosinophilic secretions. The glands of adenofibroma are abundant, larger, and tubular and more variable in size than the uniform tubules of minor sex cord elements [1]. Comparisons of key histologic features to the above mentioned differential diagnosis has been discussed in Table 1.

**Table 1:** Comparison of key histologic features to the mentioned differential diagnosis

**Histologic features**	**Fibroma with minor sex cord elements**	**Ovarian Fibromatosis**	**Brenner tumor**	**Adenofibroma**
**Spindled cells in fascicles & whorls**	Present	Present	Present	Present
**Collagen**	Variable	Abundant	variable	variable
**Normal ovarian follicles**	Replaced by fibrous stroma	Preserved	absent	absent
**Small nests of undifferentiated sex cord type cells**	Present (<10% of tumor area)	absent	absent	absent
**Edema**	absent	Present	absent	absent
**Epithelial nests of transitional/mucinous cells**	absent	absent	Present	absent

In 1983, Young and Scully reported seven cases of fibromatous tumors of the ovary, of which only five cases were ovarian fibroma with minor sex cord elements [1]. The rest of the two cases showed steroid hormone type of cells. These tumors were classified as luteinized thecoma and stromal-leydig cell tumor with minor sex cord elements. Zhang et al in 1982 studied 50 cases of luteinized thecomas and stromal Leydig cell tumors [2]. They found the presence of sex cord elements with granulosa cell morphology in only two of 50 cases. Similarly Lee et al reported a case of fibrothecoma with minor sex cord elements showing focal fibrosarcomatous change [7]. The criteria used for the diagnosis of fibrosarcomatous change were increased mitoses, hypercellularity and mild to moderate nuclear atypia. The benign fibrothecomatous areas showed minor sex cord elements with granulosa cell and steroid cell morphology. These islands of cells expressed inhibin immunohistochemically. The uterus showed multifocal endometrial adenocarcinoma of endometrioid type (FIGO grade 1) in a background of diffuse complex atypical hyperplasia. The presence of endometrial hyperplasia and cancer suggested hormone production by the ovarian tumor. Yang et al reported a case of mucinous cyst adenoma coexisting with stromal tumor with minor sex cord elements [4]. Similarly minor sex cord components representing less than 10% of the tumor area have also been described in association with testicular fibromas [8]. The fibromatous areas in these cases expressed immunoreactivity for Vimentin and SMA and the minor sex cord component expressed cytokeratin, MIC 2 and inhibin. Similarly, the spindled cells in the present case were immunoreactive for vimentin and SMA

These tumors like, fibromas are thought to behave in a benign fashion and have a good prognosis.

## Conclusion

Fibroma with minor sex cord element is a distinct clinicopathologic entity among unclassified sex cord stromal tumors and awareness of the occurrence of this uncommon entity in a normal sized ovary is important for a practicing pathologist.
